# Microglial Activation Under Hypoxic Conditions in Early Alzheimer's Disease: Can Natural SIRT1 Activators Be Therapeutic Allies in the Inflammation–Energy Axis?

**DOI:** 10.1002/ptr.70317

**Published:** 2026-03-28

**Authors:** Sara Merlo, Cristiana Lucia Rita Lipari, Aurora Patti, Maria Angela Sortino

**Affiliations:** ^1^ Department of Drug and Health Sciences University of Catania Catania Italy; ^2^ Department of Biomedical and Biotechnological Sciences University of Catania Catania Italy

**Keywords:** Alzheimer's disease, hypoxia, Mediterranean diet, mitochondria, natural SIRT1‐inducers, neuroinflammation

## Abstract

Alzheimer's disease (AD) is a progressive neurodegenerative condition characterized by a preclinical stage that typically lasts for decades. Early on during this time, microglia react to pathological changes and become protective and even transiently delay neurodegeneration. In contrast, microglia later acquire the typical pro‐inflammatory features that contribute to neurodegeneration in advanced disease. Such decades‐long time frame is marked by a significant vulnerability to any event able to tip the balance toward inflammatory microglia. Increasing evidence suggests that early life hypoxic events could be risk factors for AD by acting as early triggers of microglial phenotypic transition, especially affecting mitochondrial functions and energy balance. The NAD+‐dependent deacetylase SIRT1 could be a valuable target in this context for its anti‐inflammatory and anti‐aging functions, which include direct modulation of mitochondrial homeostasis. Many natural compounds enriched in Mediterranean diet foods, such as citrus and olives, could prove protective by inducing SIRT1 and therefore both reducing neuroinflammation and promoting mitochondrial health. This would be in line with data reporting how the type of diet can affect AD risk. Based on these premises, we here discuss the links between hypoxia, neuroinflammation, energy imbalance and mitochondrial alterations. We will describe how SIRT1 can represent a key molecular link and an appealing target to harness microglial neuroprotective potential as a preventive strategy during the key time frame of pre‐symptomatic AD. We conclude with a brief summary of the up‐to‐date evidence on the benefits of natural SIRT1 inducers that act on the inflammation‐energy axis.

**Abbreviations**
AD: Alzheimer's disease
APP: amyloid protein precursor
ATP: adenosine triphosphate
HMGB1: high mobility group box 1
IL‐6: interleukin‐6
LD: lipid droplet
LPS: lipopolysaccharide
MCI: mild cognitive impairment
MeDi: Mediterranean diet
MEL: melatonin
NAD+: nicotinamide adenine dinucleotide
NAR: naringenin
NF‐kB: nuclear factor kappa‐light‐chain‐enhancer of activated B cells
NRF1: nuclear respiratory factor 1
OXPHOS: oxidative phosphorylation
RSV: resveratrol
SIRT1: silent information regulator sirtuin 1
TGF‐β: transforming growth factor‐β
TNF‐α: tumor necrosis factor‐α
TREM2: triggering receptor expressed on myeloid cells 2
WT: wild type

## 1 Introduction

**FIGURE 1:**
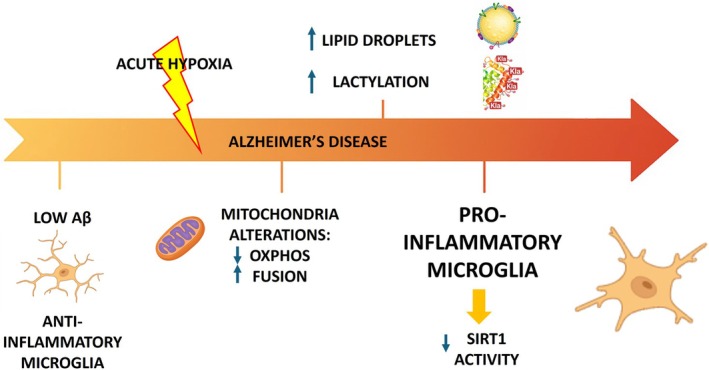
Acute hypoxia as a triggering event accelerating microglial reprogramming in AD. In early stages of AD, low levels of β‐amyloid (Aβ) are associated with an anti‐inflammatory microglial profile. Acute hypoxic insults act as a pivotal triggering event, promoting mitochondria alterations, lipid droplets accumulation, and an increase in protein lysine lactylation (Kla). This transition is associated with reduced SIRT1 activity and a switch towards an inflammatory microglial phenotype.

Alzheimer's disease (AD) is a neurodegenerative disorder of the elderly characterized by progressive amnestic, cognitive and functional decline (Walsh and Selkoe 2004). Today AD is a leading cause of death worldwide and a major socio‐economic burden, for its incidence has been growing and destined to increase along with the geriatric population (Chen et al. 2024; Xiaopeng et al. 2025). Extracellular plaques formed by aggregated β‐amyloid peptide (Aβ) and intracellular tangles of oligo‐phospho‐Tau are the histological hallmarks in the AD brain (Long and Holtzman 2019). Aβ in particular, in its different aggregation forms, has long been considered the main pathological culprit in AD (Selkoe and Hardy 2016), offering a seemingly straightforward pharmacological target for intervention. However, the road to effective therapeutics has been far from easy, for AD pathogenesis is complex and multifactorial. Older therapeutical options only offer symptomatic relief (Fox et al. 2025), while the recently FDA‐ and EMA‐approved anti‐Aβ drugs achieve only slight delays in cognitive decline and their actual benefit is still a matter of debate (Rafii and Aisen 2025). One of the most striking aspects of AD is the decades‐long lag between the initial molecular/cellular triggers and the symptomatic onset that allows for clinical diagnosis (Walsh and Selkoe 2007; Selkoe and Hardy 2016; Heneka, van der Flier, et al. 2025). As a result, AD patients remain unaware of the processes already taking place in their brains in those very years when exposure to different risk or protection factors (mainly comorbidities and life style), can decide the fate of disease progression (Figure 1). Focus on this time frame is then critical both for identifying risk factors and to develop efficient preventive approaches.

In the last few years, hypoxia has drawn attention for its involvement as a risk factor in AD. Hypoxia negatively impacts cellular metabolism and mitochondrial health and can promote microglial‐sustained inflammation (Lu et al. 2006; Orihuela et al. 2016; Zhang et al. 2017). The occurrence of hypoxic events could thus worsen AD progression specifically by acting on microglial energetics and activation (Tao et al. 2024). In this scenario, the NAD+‐dependent deacetylase SIRT1 appears of particular interest as it holds at once anti‐inflammatory activity, metabolic homeostatic functions, and mitochondrial health‐promoting functions (Figure 1) (Kauppinen et al. 2013; Vachharajani et al. 2016; Xu et al. 2018; Singh and Ubaid 2020). The diet can offer a wide variety of foods naturally rich in substances able to promote SIRT1 signaling (Iside et al. 2020; Akan et al. 2022). Many of these are present in the Mediterranean diet (MeDi), which is characterized by abundant consumption of fruits, vegetables, legumes, whole grains, fish, and olive oil (Russo et al. 2014; Kiani et al. 2022). Accordingly, MeDi has been recognized to be a protective factor in AD (Russo et al. 2014; Schwingshackl et al. 2020; DiNicolantonio et al. 2022; Arora et al. 2023; Grant and Blake 2023; Nucci et al. 2024).

With these considerations in mind, we here present the most up‐to‐date research linking hypoxia, neuroinflammation, SIRT1, and SIRT1‐inducing Mediterranean dietary compounds, offering a unified view on this keynote topic.

## 2 Alzheimer's Disease and the Janus‐Faced Nature of Microglia

In the setting of pre‐symptomatic AD, microglia‐sustained neuroinflammation has emerged as an extremely relevant factor (Heneka, van der Flier, et al. 2025). By definition, inflammation represents a transient response acting as a first line of defense to restore compromised homeostasis (Newcombe et al. 2018; Galea and Graeber 2023). However, it is now well‐established that a gradual accumulation of unresolved inflammatory reactions can lead to a harmful chronicization of the process (Fan et al. 2017). This seems to occur in AD, where a dual peak of microglial activation has been described, promoting protective responses during pre‐MCI stages but later switching towards ineffective or even detrimental features (Whittington et al. 2017; He et al. 2025). In advanced AD, this translates into a general loss of microglial protective functions, with changes in energy status and metabolism, mitochondrial dysfunctions (Wu et al. 2025; Yang, Liang, et al. 2025), inability to clear Aβ deposits (Lepiarz‐Raba et al. 2023), aberrant synaptic pruning (Tzioras et al. 2023) and overall neurotoxic effects (Azmal et al. 2025). Such dualism is unquestionably a simplified description of a much more complex picture, as microglial phenotypes seem to fall within a continuum between protective/anti‐inflammatory versus detrimental/chronically inflammatory states, that sometimes even show overlapping gene expression (Town et al. 2005; Merlo, Spampinato, et al. 2020). In the last few years, at least six restricted populations with specific genetic signatures have been identified in the AD brain. In a recent study, using a multiomic approach in human brain‐derived iPSCs activated into the different phenotypes, the shared expression of one or more key genes across different programs was confirmed (McQuade, Castillo, et al. 2025; McQuade, Mishra, et al. 2025).

### 2.1 Disease‐Associated Microglia (DAM)

The first identified AD‐associated phenotype was defined as DAM, detected in association with amyloid plaques both in animal and human AD brains. These cells are characterized by marked protective clearing functions (Keren‐Shaul et al. 2017) and work as sensors‐responders to CNS damage through neurodegeneration‐associated molecular patterns and TREM2 signaling (Deczkowska et al. 2018). In agreement, pathways that restrain microglia in their homeostatic phenotype have been suggested to hinder the switch towards the protective DAM phenotype. TGF‐β1‐mediated induction of the immune checkpoint molecule TIM3 (encoded by HAVCR2 in microglia) was shown to promote microglial homeostasis, while its genetic deletion was associated with an increased DAM phenotype. Furthermore, microglia‐targeted deletion of HAVCR2 in the 5xFAD mouse model was shown to reduce amyloid‐β accumulation and cognitive impairment (Kimura et al. 2025).

### 2.2 Lipid Droplet‐Accumulating Microglia (LDAM) and Interferon‐Responsive Microglia (IRM)

On the opposite, dysfunctional states that impair microglial defensive abilities have been identified, namely LDAM and IRM. LDAM are associated with both aging and the ApoE4 genotype (Marschallinger et al. 2020; Haney et al. 2024; Lu et al. 2025). Microglial accumulation of lipid droplets (LD) was selectively increased near Aβ plaques both in human brains and in the 5xFAD mouse animal model. In agreement, the enzyme diacylglycerol O‐acyltransferase 2 (DGAT2), which converts free fatty acids in triglycerides, was upregulated by Aβ while DGAT2 targeting restored phagocytosis by microglia (Prakash et al. 2025). In another study, carried out on human induced pluripotent stem cell‐derived microglia, fibrillary Aβ was shown to upregulate the expression of the enzyme acyl‐CoA synthetase long‐chain family member 1 (*ACSL1*), another key enzyme involved in LD biogenesis (Haney et al. 2024). Conversely, TREM2 was found to be a key player in limiting LDAM appearance, acting as a sensor and regulator of homeostatic lipid metabolism (Marschallinger et al. 2020; Zhang et al. 2025). In AD, TREM2 was shown to support the metabolic fitness in microglial cells by promoting energetic and bio synthetic metabolism (Ulland et al. 2017). In AD patients and mouse models, lack of TREM2 function was in fact shown to be associated with defective mammalian target of rapamycin (mTOR) signaling, which led to increased autophagy. In agreement, restoration of these pathways increased microglial clustering around amyloid plaques, ameliorated neuronal dystrophy next to plaques and diminished autophagy in TREM2‐deficient AD mice (Ulland et al. 2017). Of great interest are the findings by Bosch et al. (2020) regarding the role of LDs in innate immunity, acting as molecular activators of metabolic reprogramming in macrophages, in response to stress signals in vitro. In particular, after LPS exposure, perilipin (PLIN) 5 downregulation mediated the metabolic disconnection between LDs and mitochondria, while PLIN2 upregulation favored the accumulation of inflammatory factors around LDs also through a direct physical interaction (Bosch et al. 2020). If translated to microglia, this mechanism could have direct repercussions on metabolic reprogramming and on the trajectory of microglial activation in AD, suggesting PLINs as novel targets for therapeutic intervention.

Finally, IRM are characterized by an elevated expression of interferon‐induced genes even in the absence of viral infections and were associated with aging and various disease states, including AD (McQuade, Castillo, et al. 2025). A key pathway in the IRM program is cGAS‐STING signaling. In a study carried out on 5xFAD mice, activation of such pathway contributed to AD pathology, while its genetic ablation or pharmacological blockade ameliorated pathogenesis and cognitive impairment (Xie et al. 2023). Moreover, IRM were found in association with neuritic plaques in post‐mortem AD patients' brains (Roy et al. 2020).

The existence of a specific time‐frame when microglia can be anti‐inflammatory and neuroprotective intuitively suggests both positive and negative implications. On one side microglia can be targeted to sustain and prolong their beneficial features, while on the other they can be impacted by external factors that can anticipate their transition to unhelpful or detrimental phenotypes.

Current efforts are dedicated to unraveling the complex transcriptional regulation driving differential activation to identify new targets for microglial reprogramming strategies. As mentioned, this is particularly important in early stages which have been less characterized. While the late inflammatory events related to advanced AD have been more detailed and many potential targets identified (Melchiorri et al. 2023; Kamila et al. 2025), the past limitations in early diagnosis have hindered progress in the field. The advent of fluid biomarkers in the next few years will optimistically enable preventive approaches for at‐risk subjects (Heneka, Gauthier, et al. 2025).

## 3 Hypoxia and AD: A Harmful Connection

The term hypoxia refers to the lowering of oxygen availability at the cellular level, a condition that can originate from a number of causes and is either transient or chronic. Transient hypoxia consists in a temporary lack of adequate oxygen supply to the cells due to hypoventilation or hypoperfusion, commonly caused by respiratory/cardiovascular issues or low environmental oxygen, as for example at high altitudes (Busl and Greer 2010; Burtscher et al. 2012). Oxidative metabolism by mitochondria represents an evolutionary success that improved ATP production, enabling the support of higher brain functions that require great amounts of readily available energy. Accordingly, the brain is the largest consumer of bodily oxygen among all organs and neuronal cells cannot endure prolonged hypoxia without irreversible damage and death (Erecinska and Silver 2001).

The connection between hypoxia and AD in the past has been largely addressed from a neuronocentric point of view, evidencing neuronal vulnerability and pro‐amyloid and tau effects (Hassan and Chen 2021). More recently, however, as the role of the neuroinflammation‐energy axis in both AD and hypoxia has become clearer, the latter has been implicated as a potential risk factor for AD. The focus in this regard is currently directed towards metabolic reprogramming and impairment of lipid metabolism, which leads to LD accumulation.

### 3.1 Metabolic Reprogramming in Microglia

During microglial inflammation, metabolic reprogramming entails a shift toward glycolytic ATP production, under both aerobic and anaerobic conditions, favoring speed of energy delivery over total energetic yield (Lauro and Limatola 2020). An immediate consequence is the build‐up of lactate, which was very recently recognized as a novel inflammatory signaling molecule and gene expression modulator, rather than just a mere metabolic by‐product. Excess intracellular lactate is covalently bound to lysine residues on both histone and non‐histone proteins, a process defined as *lactylation*. The increase of lactylated proteins was associated with pro‐inflammatory gene expression in microglia and has been observed in neurodegenerative conditions as well as during hypoxia (Gao et al. 2025; Han et al. 2025; Liao et al. 2025; Wang et al. 2025; Yang, Mo, et al. 2025). It is worth noting that class III histone deacetylases SIRT1 and SIRT3 display de‐lactylating activity, which could take part in their anti‐inflammatory action (Moreno‐Yruela et al. 2022).

Metabolic lipid alterations described during hypoxic inflammation involve the switch to LDAM phenotype. In a recent study using in vitro and in vivo models of stroke, microglia were shown to accumulate LD and release pro‐inflammatory cues, with ensuing brain injury (Wei, Lattau, et al. 2024). In another study, TREM2 signaling was shown to prevent microglial LD accumulation via TGF‐β1 release after ischemic injury both in primary cultures and in vivo models, with a protective effect (Wei, Zhang, et al. 2024). This could suggest a dual type of post‐ischemic microglial activation, compatible with the protective‐to‐detrimental change aforementioned for AD. The role of hypoxia in the impairment of lipid metabolism was further corroborated by data from in vivo models of high altitude exposure: inflamed and IL‐1β‐producing LDAM appeared 1 week after the insult and directly correlated with cognitive impairment (Fan et al. 2025).

Strikingly, even antenatal/perinatal hypoxic events were shown to favor an increased vulnerability to AD in later life, by imparting life‐lasting microglial inflammatory signatures (Shen et al. 2020; Arruda et al. 2025). In a study using the 5xFAD mouse model, antenatal hypoxia accelerated the onset of cognitive decline, with increased microgliosis compared to that observed in the hypoxic WT (Shen et al. 2020).

Finally, the ability of hypoxia to reverse the microglial protective phenotype in a simplified “early AD” in vitro model has been recently proven (Merlo et al. 2018; Caruso et al. 2021). This involved a reduction in oxygen consumption upon re‐oxygenation and significant changes in mitochondrial morphology (Lipari et al. 2025).

To summarize, hypoxic events can occur early during asymptomatic AD, later during symptomatic disease, or even before any of the AD‐specific cellular events have yet taken place, and all find a common target in the microglial inflammation‐energy axis.

## 4 Mitochondria at the Crossroads of Hypoxia and AD Pathogenesis

Mitochondria represent the energy factories of the cell and play a central role in energy production, as well as in redox balance, calcium homeostasis and apoptotic signaling. Mitochondrial activity consists of various biochemical steps that metabolize substrates to produce ATP, the main energy currency of the cell. Mitochondria exploit molecular oxygen as the final acceptor in the electron transport chain, during the process of oxidative phosphorylation (OXPHOS), producing an amount of energy that is significantly higher compared to that generated by glycolytic metabolism, which does not require oxygen (Lauro and Limatola 2020). Notably, mitochondria are specialized to dynamically adapt to specific cellular energetic requirements by fusion or fission events aimed at functional optimization, mitophagy to discard damaged organelles, and biogenesis to generate new ones. These activities are overall referred to as mitochondrial quality control (MQC) (Li et al. 2022; Liu, Wang, et al. 2025).

### 4.1 Dynamic Changes in Microglial Mitochondria and AD

As said, the human brain is one of the most oxygen‐dependent organs; it constitutes on average 2% of the total body weight, but utilizes up to 25% of total body glucose and 20% of body oxygen in the resting awake state (Zhu et al. 2018). As a consequence, the brain is highly vulnerable to both oxygen shortage and to impaired energy metabolism due to mitochondrial damage of different origin, from disease to environmental causes, linked to genetic mutations or aging (Wang, Shi, et al. 2024; Belenichev et al. 2025). Not surprisingly, mitochondria are both targets and mediators of neuroinflammation and their dysfunction is increasingly recognized not merely as a downstream consequence, but as a primary driver in the pathogenesis of many neurodegenerative conditions, including AD and hypoxic/ischemic injuries (Alves et al. 2025; D'Alessandro et al. 2025). When hypoxic events are layered with AD pathogenesis, mitochondrial dynamics can be pictured as modifiable risk factors for disease progression. Accordingly, the correlation between mitochondrial stress, AD and hypoxia has been intensively addressed in the last few years.

Several examples confirm that, in AD, perturbations in microglial MQC are responsible for locking microglia in dysfunctional/maladaptive states, where energy imbalance amplifies oxidative stress and impairs vital ATP‐dependent processes.

From the point of view of microglial functions, mitochondrial health is crucial for sustaining energy intensive processes such as phagocytosis, motility, cytokine production, and maintenance of redox homeostasis (Baik et al. 2019; Li et al. 2022; Shokr 2025; Yang, Liang, et al. 2025). In an extensive study investigating the link between impaired MQC and AD, it was shown that mitophagy was impaired in the hippocampus of AD patients, in human iPSCs‐derived neurons and in animal AD models. Pharmacological rescue of mitophagy could revert AD pathology and memory impairment in APP/PS1 mice by enhancing microglial phagocytic functions and suppressing inflammation (Fang et al. 2019). In another study, the translocator protein TSPO and the mitochondrial hexokinase (HK)‐2 were shown to concurrently regulate the glycolytic switch, impacting phagocytosis in AD mice and primary microglial cultures exposed to Aβ. In these models, while TSPO favored OXPHOS and sustained phagocytosis, binding of HK‐2 to mitochondria promoted glycolysis and reduced phagocytic abilities. Strikingly, Aβ exposure directly increased HK‐mitochondria binding in cultured microglia (Fairley et al. 2023). It is also important to mention that Aβ can enter the cell and directly affect mitochondria by interaction with outer membrane translocases (e.g., TOM complexes) that import the peptide within the organelle with consequent disruption of its functions (Pinho et al. 2014). In microglia, this has been associated with reduced phagocytosis. Finally, environmental factors can contribute to AD‐related mitochondrial dysfunctions as exemplified by a very recent study exploring the effects of copper overload. Both in vitro and in vivo AD settings, copper selectively localized in mitochondria where it mediated inflammasome activation and altered cholesterol metabolism, amplifying Aβ‐related inflammation and reducing Aβ‐clearance (Zubillaga et al. 2025).

### 4.2 Hypoxia and Microglial Mitochondrial Function: Impact on AD

Several lines of evidence from in vitro and in vivo studies support the notion that chronic, intermittent, and acute hypoxic episodes can all impact AD by precipitating mitochondrial dysfunction (Liu et al. 2023; Sharma et al. 2025). As mentioned above, a direct involvement of mitochondrial alterations in the effects of mild acute hypoxia in HMC3 human microglia has been identified (Lipari et al. 2025). The M2‐like phenotype induced by early Aβ exposure to mimic early AD stages was reversed after exposure to hypoxia. In addition, oxygen consumption was reduced, and so was the number of mitochondria, which, however, became larger in size. These results suggested a metabolic rearrangement promoting mitochondrial fusion to face the reduction in oxygen availability, which, however, accelerated microglial inflammation (Lipari et al. 2025). More literature is available regarding the effects of hypoxia in advanced AD. Recently, the correlation between Aβ‐induced microglial activation and HIF‐1 signaling was explored in an extensive study including the APP/PSEN1 mouse model, primary cultures from the same transgenic mouse, and human post‐mortem AD samples. Results paradoxically showed the selective upregulation in plaque‐associated microglia of both the glycolytic trigger HIF‐1 and of several OXPHOS‐related genes (March‐Diaz et al. 2021). In particular, Aβ‐associated microglia in the mouse model displayed elongated mitochondria, suggestive of an attempt to optimize aerobic respiration and prevent the glycolytic shift (Gomes and Scorrano 2011; March‐Diaz et al. 2021; Lipari et al. 2025). Such compromised metabolism translated in a reduced ability of microglia to cluster around plaques and to clear Aβ (March‐Diaz et al. 2021). In a different study, transcriptomic analyses in 5xFAD mice exposed to chronic intermittent hypoxia revealed a unique signature pointing to microglial alterations in OXPHOS (Marino et al. 2025). In agreement with the anti‐inflammatory role of OXPHOS as opposed to an enhanced glycolytic metabolism, the administration of hyperbaric oxygen could rebalance MQC, reflecting on microglia with the reversion to a resting surveillance phenotype in 5xFAD transgenic mice (Yao et al. 2026). Altogether, these data confirm that mitochondria stand at the crossroads of hypoxia and AD and could account for the former being a risk factor for the disease.

### 4.3 Transfer of Healthy Mitochondria From Microglia to Neurons: An Intriguing Mechanism for Survival

**FIGURE 2:**
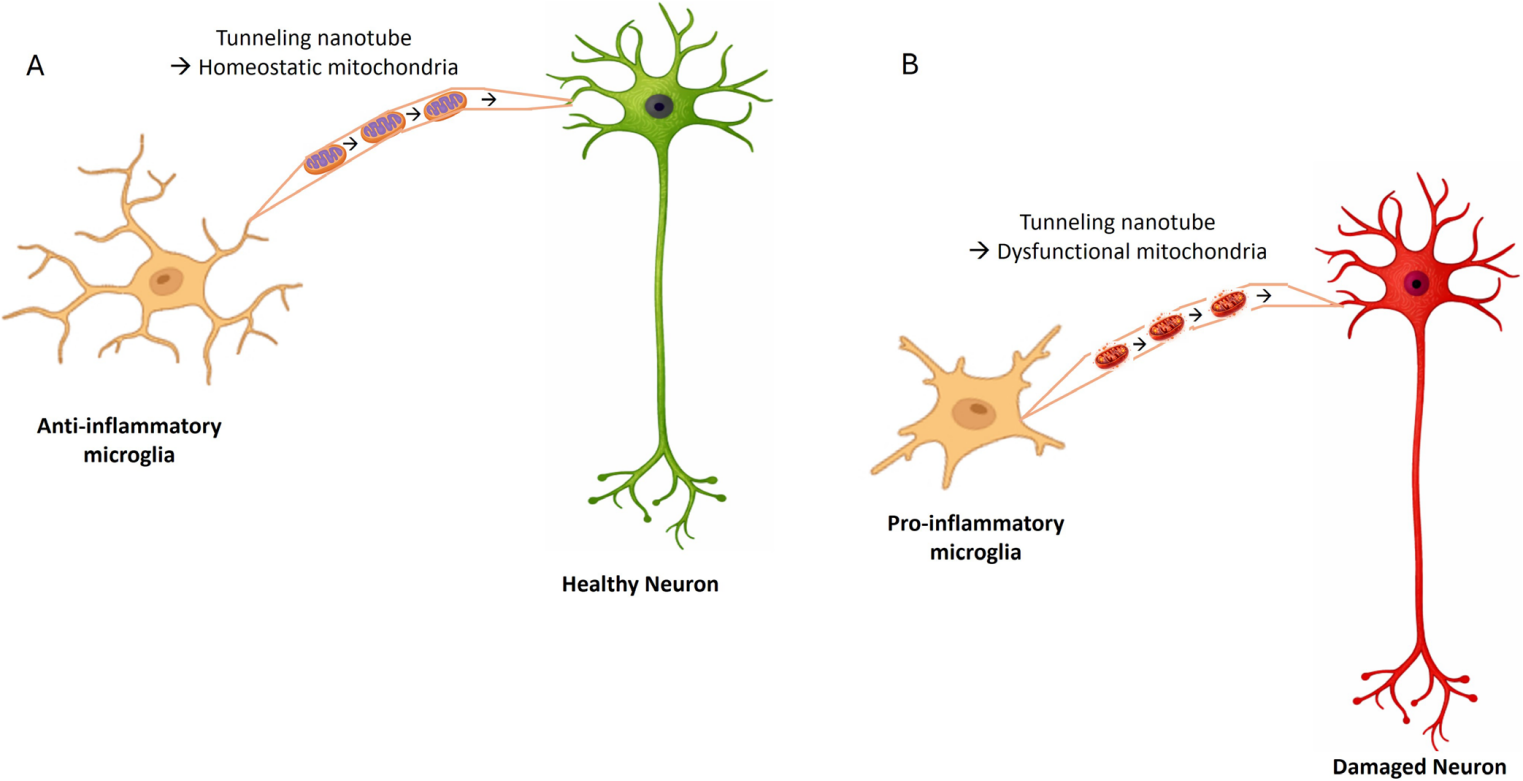
Microglia–neuron mitochondrial transfer. Anti‐inflammatory microglia can support neuronal homeostasis through tunneling nanotube‐mediated transfer of functional mitochondria (A), whereas pro‐inflammatory microglia could transfer dysfunctional mitochondria (B), contributing to neuronal degeneration.

It is finally worth citing the growing literature regarding a physical communication between microglia and neurons through tunneling nanotube structures (TNTs) and extracellular vesicles (Rustom et al. 2004; Chen et al. 2020), which allow effective mitochondrial transfer from one cell type to the other (Pereira‐Santos et al. 2024; Wei, Du, et al. 2024). Organelle transfer can clearly have a profound impact in the course of AD and has been shown to exert both positive and negative outcomes, depending on the condition of transferred mitochondria (Figure 2). In neuronal‐microglia co‐cultures, exposed to alpha‐synuclein alone or with tau in AD‐like settings, healthy mitochondria were transferred from microglia to damaged neurons, improving neuronal health and reducing oxidative stress (Chakraborty et al. 2023; Scheiblich et al. 2024). Interestingly, AD‐relevant mutations in leucine‐rich repeat kinase 2 (LRRK2) or TREM2 impaired this process (Scheiblich et al. 2024). On the other hand, an in vitro study on murine BV2 microglia exposed to oxygen–glucose deprivation showed ongoing mitochondrial fission, with subsequent release of damaged mitochondria that could be acquired and fused with neuronal ones. This resulted in impaired mitochondria‐mediated neuronal death and exacerbation of the injury (Liu et al. 2022). Currently, the development of next‐generation cell therapies based on mitochondrial transfer is an exciting and active new area of research (Huang et al. 2022; Meng et al. 2025).

## 5 Bridging Energetics and Inflammation: the Central Role of SIRT1

SIRTs constitute a family of seven highly conserved NAD+‐dependent deacetylases involved in the control of metabolism and longevity. SIRTs are subjected to a very strict regulation involving transcriptional, post‐transcriptional and post‐translational modifications (Satoh et al. 2017; Xu et al. 2023). In addition, their activity depends on the availability of adequate levels of NAD+, which serves as a coenzyme and is a key signaling molecule regulating intermediary metabolism (Verdin 2015; Xu et al. 2023). Among sirtuins, SIRT1 has gained particular attention over the years, with a plethora of literature demonstrating its beneficial roles across multiple organs and diseases, including CNS hypoxic injury and AD (Chojdak‐Lukasiewicz et al. 2022).

**FIGURE 3:**
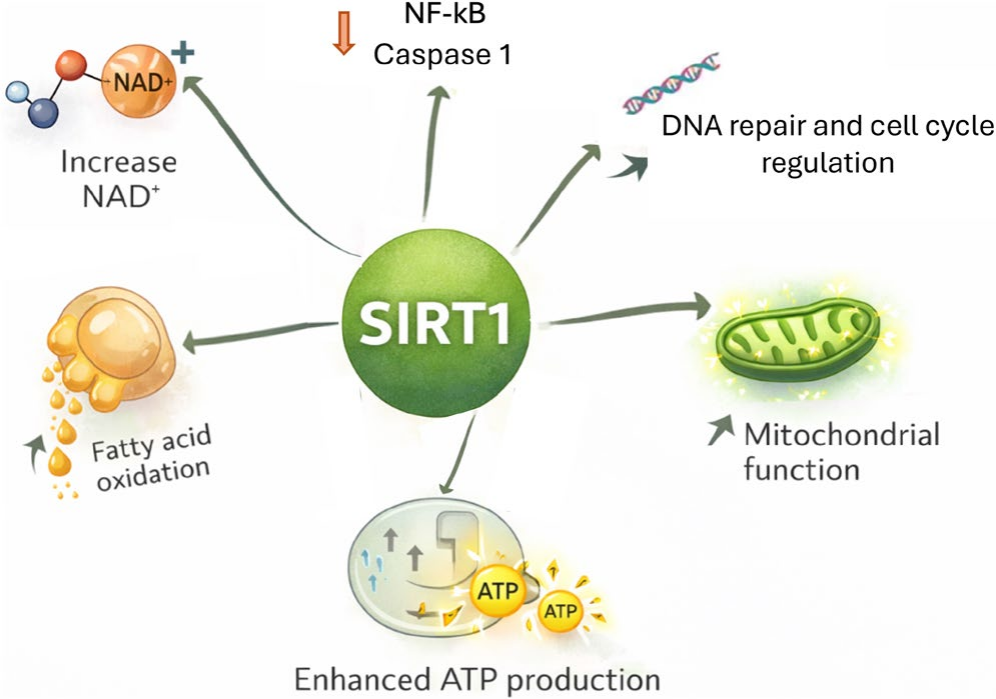
Multiple effects of SIRT1. Increased SIRT1 activity supports energy metabolism and mitochondrial function, enhances fatty acid oxidation, promotes DNA repair and down‐regulates pro‐inflammatory signaling pathways (e.g., NF‐kB, Caspase 1).

SIRT1 is endowed with strong anti‐aging, anti‐inflammatory and anti‐oxidant actions (Figure 3) (Jiao and Gong 2020). These effects are accounted for by both the large number of genes that are modulated by SIRT1‐mediated histone‐deacetylation and the many proteins that are direct SIRT1 targets, as shown in several tissues, including the brain. Strikingly, the list includes proteins that are relevant to hypoxia, inflammation, mitochondrial metabolism and MQC, as HIF‐1, NF‐kB, HMGB1, PGC‐1α Mitofusin (MFN) 2, DRP1, and Forkhead box O (FOXO), just to name a few (Yang et al. 2022; Xu et al. 2023; Link and Ferreira 2025). Most metabolic enzymes expressed in mitochondria are indeed modulated by acetylation/deacetylation and sirtuins have been shown in several tissues to regulate mitochondria and mitophagy (Zhao et al. 2010; Wan et al. 2022; Chaqour et al. 2025; Lagunas‐Rangel 2025). SIRT1‐dependent deacetylation is sensitive to the NAD+/lactate balance and it is inhibited by both reduced NAD+ availability and elevated lactate levels, suggesting that strategies to sustain SIRT1 could prove beneficial against the challenges that impair mitochondrial OXPHOS and induce a glycolytic switch, increased lactate production and inflammation. Moreover, SIRT1 is connected with another key bioenergetic regulator, the AMP‐activated protein kinase (AMPK), by a reciprocal regulation and shared targets (Ruderman et al. 2010), as recently reviewed (Chen et al. 2025; Guan et al. 2025).

It is worth noting that while deacetylation is the main enzymatic function of sirtuins, they can display additional mechanism that could contribute to SIRT1 anti‐inflammatory actions in the context of hypoxia and AD, preventing the effects of metabolic reprogramming that are mediated by increased lactate. Another interesting finding comes from a recent study in hepatocytes where a direct involvement of SIRT1 in the formation of TNTs has been suggested to impact on the transfer of healthy mitochondria between cells (Wang, Zhu, and Zhang 2024). This mechanism is proposed to depend on the interplay between SIRT1 and cortactin/F‐actin, and finds support in the literature linking SIRT1 to cytoskeletal modulations in different tissues like muscle, kidney and the cornea (Nakatani and Inagi 2016; Iwahara et al. 2022; Lin et al. 2022). Again, should this mechanism be proven also in the CNS, it would further corroborate the critical role of SIRT1 in promoting beneficial effects in the energy‐inflammation axis.

Finally, SIRT1 is directly implicated in balancing lipid storage and mitochondrial functionality through a direct interaction with the LD‐associated proteins. PLIN5 was shown to interact with SIRT1 and the PGC1‐α pathway to synchronize lipolysis with mitochondrial activity (Gallardo‐Montejano et al. 2016). Moreover, it activates SIRT1, promoting autophagy and preventing inflammation induced by LPS and palmitate in hepatocytes (Zhang et al. 2020).

Overall, it appears evident that SIRT1 holds the potential to modify risks connected to hypoxia‐induced metabolic alterations that activate microglia in the context of AD. As such, SIRT1 is a highly relevant therapeutic target and ways to support its function ideally represent successful strategies.

## 6 SIRT1 Activators in the Mediterranean Diet: A Natural Boost for Cellular Metabolism, Mitochondrial Health, and Protective Microglia

As mentioned, natural SIRT1 activators are abundant in a variety of MeDi foods which can be referred to as “sirtfoods” (Iside et al. 2020; Akan et al. 2022). The largest class of sirtfoods consists of polyphenols, of both flavonoid and non‐flavonoid nature (Akan et al. 2022). Most polyphenols can cross the blood‐brain barrier and therefore directly reach brain cell targets (Figueira et al. 2021). Accordingly, a recent systematic literature review has highlighted the pivotal role of the MeDi in mitigating the risk of age‐related cognitive decline, dementia and AD (Fekete et al. 2025). The beneficial effects of MeDi contrast with the *Western diet*, which is characterized by a greater consumption of saturated fats, simple carbohydrates, and high sodium content, all associated with pro‐inflammatory outcomes (Hoscheidt et al. 2022). The long‐term effects of MeDi‐derived nutrients on cognitive and inflammatory biomarkers in AD were recently evaluated in a large clinical study comparing patients to healthy controls. Results suggested a strong association of the diet with cognitive preservation and lower blood and CSF levels of inflammatory markers, such as IL‐6 and TNFα. Importantly, the benefits were proportional to effective diet adherence (Liu, Yang, et al. 2025).

**FIGURE 4:**
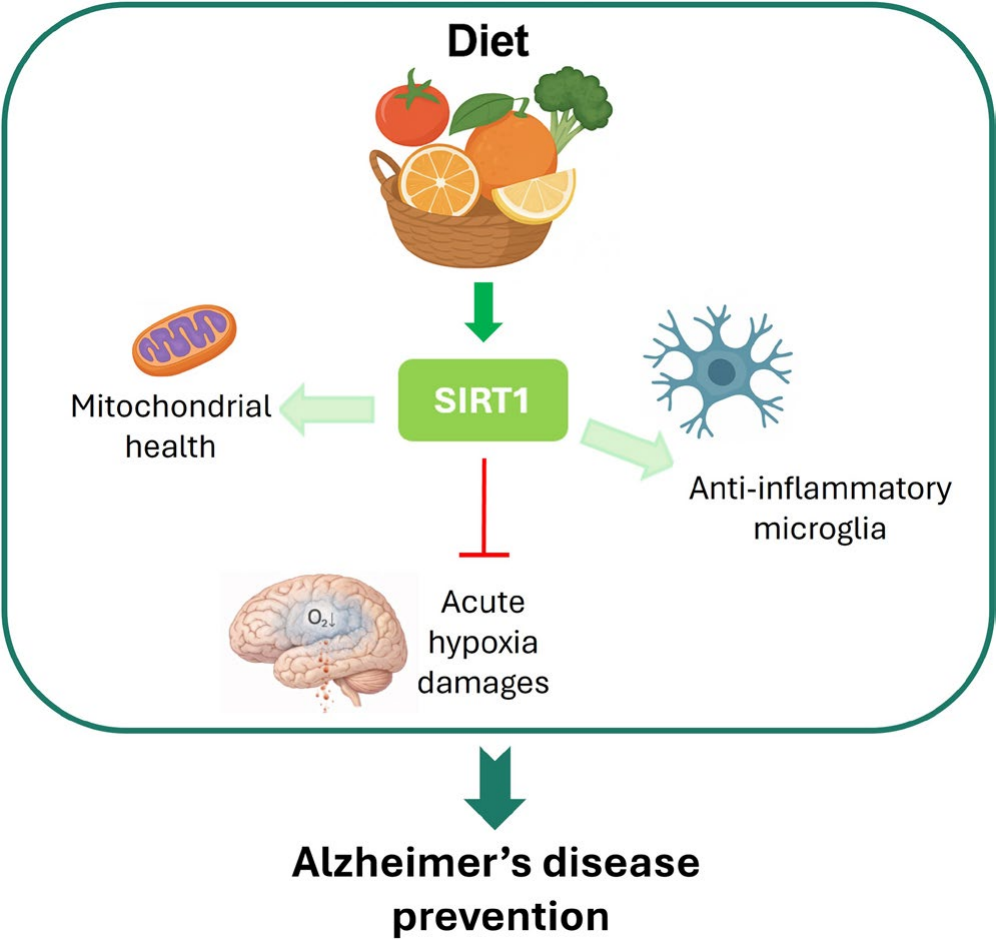
Dietary activation of SIRT1 as a protective strategy in AD. Natural compounds enriched in the Mediterranean diet increase SIRT1 levels and activity, promoting anti‐inflammatory microglial polarization, mitochondrial health, and limiting hypoxia‐induced cellular damage.

The role of MeDi‐provided natural substances in promoting microglial‐sustained neuroprotection vs. inflammatory activation has been amply established in preclinical studies, while actual clinical efficacy yet needs to be confirmed. Previous reviews have covered those studies investigating the anti‐inflammatory properties of MeDi compounds (Hornedo‐Ortega et al. 2018) and the benefits of natural substances specifically acting as SIRT1 modulators in AD (Figure 4) (Zhang and Tang 2023). Here, we will focus specifically on substances from the MeDi that are endowed with SIRT1‐inducing activity and that modulate the inflammation‐energy axis. These are of most interest in the context of hypoxia occurring in the course of AD since, by positively affecting microglia exposed to hypoxia, they can modify the risk for AD progression.

### 6.1 Resveratrol

Resveratrol (3,5,4′‐trihydroxystilbene) (RSV) belongs to the class of stilbenes and is one of the most studied natural SIRT1‐inducing compounds. RSV is especially enriched in grapes, berries, pomegranate and peanuts (Akan et al. 2022). RSV induces SIRT1 expression and enhances SIRT1 enzymatic activity by allosteric stabilization of its interaction with different substrates, as from in silico evidence (Hou et al. 2016). The beneficial properties of RSV have been associated during the 90s with what has become known as *the French paradox*, that is, the unexpectedly low cardiovascular mortality rates observed in the French population from the region of Bordeaux, despite their typically high saturated fat‐diet (Bakht et al. 2025). The observation was at least in part explained by the presence of selective RSV phytocomplexes in red wine grapes grown in that area.

In general, the SIRT1‐inducing, mitochondrial restoration and protective effects of RSV in the CNS have been more studied at the neuronal level, both in preclinical and clinical studies (Zhu et al. 2025). In AD, neuroprotection by RSV has been linked to its anti‐inflammatory actions (Mathew et al. 2025; Puranik et al. 2025). The non‐neuronal effects of RSV were recently explored in a rat model of AD induced by the active Aβ fragment (25–35). After 4 weeks of intraperitoneal administration, RSV increased hippocampal SIRT1 expression while at the glial level it reduced microglia and astrocyte activation. Behavioral assessment confirmed ameliorated spatial and learning memory in RSV treated animals (Surya et al. 2025). RSV potentiation of SIRT1 was also demonstrated to be protective in in vivo and in vitro models of brain injury from neonatal hypoxia/ischemia. In vivo, RSV rescued the hippocampal expression of SIRT1, reduced the levels of inflammatory markers such as NF‐kB, IL‐1β and iNOS in neonatal hypoxic brain injury mice (Peng et al. 2022) and mitigated microglial inflammation by SIRT1‐mediated inhibition of HMGB1 signaling (Le et al. 2019). This latter mechanism was confirmed in vitro on murine microglial BV2 cells, where SIRT1 and HMGB1 were also confirmed to directly interact (Le et al. 2019). From a molecular point of view, evidence of a direct link between RSV, SIRT1 and the energy axis comes from studies on different models, where mitochondrial function and metabolic homeostasis were induced by RSV mainly through the SIRT1/PGC‐1α/NRF‐1/2 and SIRT1/FoxO3a/PGC‐1α axes (Shaito et al. 2023). Clinical trials to examine resveratrol supplementation in AD patients, compared to placebo, have not confirmed significant cognitive benefits but have established its safety, tolerability and penetration of the blood–brain barrier, as well as modulation of amyloid and inflammatory biomarkers (Turner et al. 2015; Moussa et al. 2017; Gu et al. 2021; Jin et al. 2023).

### 6.2 Naringenin and Naringin

Citrus fruits represent a distinctive component of the MeDi and one of the richest sources of anti‐oxidant/anti‐inflammatory compounds, including SIRT1‐inducing components such as the flavanone naringin and its aglycone derivative naringenin (NAR). Both are particularly enriched in grapefruits and present in oranges, mandarins, bergamots, and lime. In silico studies have shown that NAR can activate SIRT1 by direct interaction, in addition to inducing its expression. The involvement of SIRT1 in naringenin's anti‐inflammatory action has been extensively investigated in the cardiovascular system, where its protective effects involve the AMPK–SIRT1 cross‐talk that improves energy metabolism and sustains mitochondrial biogenesis (Testai et al. 2020; Piragine et al. 2024). NAR was shown to restore reduced levels of SIRT1 in the cardiac tissue of aged mice, an effect that correlated with the prevented increase of inflammatory markers IL‐6 and TNF‐α and of mitochondrial dysfunction (Testai et al. 2020). In an atherosclerosis mouse model, NAR was shown to attenuate vascular senescence and atherosclerosis, and mechanistic analyses in vitro on endothelial cells showed the involvement of the SIRT1‐FOXO3a‐PGC1α axis and mitochondrial protection (Wang et al. 2023). Less data is available regarding the anti‐inflammatory role on glial cells in the CNS, while more evidence has been gathered for direct neuronal actions in different pathological conditions (Shin and Shin 2024; Azizollahi et al. 2025). Recently, naringenin effects have been analyzed explicitly for their potential protective role in the context of hypoxia over AD. The direct microglial effects of NAR were investigated, confirming that in human HMC3 microglia exposed to the combined Aβ/hypoxia challenge described in the previous paragraphs, naringenin upregulated SIRT1 nuclear translocation, with downstream anti‐inflammatory effects at least in part sensitive to the selective SIRT1 inhibitor EX527. Moreover, naringenin ameliorated mitochondrial oxygen consumption and contrasted some morphological changes in a SIRT1‐dependent fashion (Lipari et al. 2025).

### 6.3 Extra Virgin Olive Oil (EVOO)

The MeDi is renowned for the use of condiments based on EVOO, which is a rich source of polyphenols (Visioli and Bernardini 2011). Although the phenolic components in olives and EVOO constitute a minor part, their presence contributes to the health benefits associated with their consumption (Romani et al. 2019). In a recent review, preclinical evidence on the neuroprotective properties of EVOO polyphenols in AD has been reported, mainly relating to neuronal effects (Wei, Li, et al. 2025). The phenolic acid oleacin (OC), glycosylated derivative oleuropein (OP), and the OP aglycone metabolite hydroxytyrosol (HT) have been shown to induce SIRT1 (Luccarini et al. 2016; Sun et al. 2019; Martinez‐Zamora 2025), and therefore hold the potential to exert the metabolic protective functions described so far for other natural substances with similar properties. The anti‐inflammatory effects of these compounds have been proven. In vitro, OP and HT both induced an anti‐inflammatory phenotype in microglial cell lines, reducing cytokines release via TREM2 activation (Leri et al. 2023). However, to date, the specific involvement of SIRT1 in combating inflammation has not been investigated. This is a field that certainly deserves attention in the future.

### 6.4 Melatonin

Melatonin (N‐acetyl‐5‐methoxytryptamine; MEL) is a human pineal hormone also produced in plants and found in several Mediterranean foods, that is, EVOO, red wine, fruits, legumes and nuts. Melatonin has a strong and well‐established relationship with SIRT1 in mitochondrial function, neuroprotection and aging (Mayo et al. 2017). MEL was able to induce SIRT1 expression in HMC3 microglia in an early AD in vitro model and delay the pro‐inflammatory transition by activation of the SIRT1‐BDNF axis (Caruso et al. 2021). This resulted in microglia‐mediated neuroprotection against Aβ‐induced synaptic and neuritic damage (Merlo et al. 2022). Additionally, MEL rescued SIRT1 expression thereby preventing inflammation both in an in vivo model of hypoxia by carotid occlusion in p7 rats and in BV2 microglia exposed to chemical hypoxia (Merlo, Luaces, et al. 2020). In the combined AD/hypoxia in vitro model, MEL accordingly prevented the lowering of SIRT1 activation and SIRT1 mediated part of MEL's anti‐inflammatory effects and the rescue of mitochondrial oxygen consumption (Lipari et al. 2025). In a study carried out in aged (18 months‐old) mice models of obstructive sleep apnea syndrome obtained by chronic intermittent hypoxia, MEL administration prevented inflammation and related cognitive decline induced by hypoxia. Specifically, MEL afforded neuroprotection by contrasting the hippocampal induction of glial markers Iba1 and GFAP, and of the key mediators of inflammation, that is, NLRP3, NF‐kB, IL‐1β, IL‐6 and TNF‐α (Wei, Zhang, et al. 2025). The involvement of SIRT1 as a mediator of MEL protection against ischemic injury has been confirmed in a very recent study focusing on astrocyte‐mediated inflammation in ischemic stroke, where MEL contrasted neuronal damage by attenuating inflammatory responses both in vivo and in vitro. Such effect depended on MEL enhancement of SIRT1 deacetylation of NF‐kB (Zhuang et al. 2025).

### 6.5 Quercetin

Quercetin [2‐(3,4‐dihydroxyphenyl)‐3,5,7‐trihydroxy‐4H‐chromen‐4‐one] is a flavonoid polyphenol contained in vegetables like onion, capers, olives, fruit like citrus and apples, commonly part of the Mediterranean cuisine. Evidences regarding SIRT1‐mediated beneficial effects of quercetin in neurodegenerative and‐age related diseases have been extensively outlined in two recent review articles (Cui et al. 2022; Zhou et al. 2025). Interestingly, in the hippocampus of rats exposed to hypoxia, quercetin was shown to restore homeostatic MQC by SIRT1‐mediated induction of PGC‐1α and MFN1 and MFN2, promoting mitochondrial fusion and biogenesis. At the same time, the expression of the fission factor DRP1 was inhibited. These events were accompanied by the partial rescue of hypoxia‐induced memory deficits (Liu et al. 2015; Zhou et al. 2025). As for other compounds, data on SIRT1's role in quercetin‐mediated anti‐inflammatory actions are still scarce but represent a promising new field of research.

### 6.6 Other Natural SIRT1 Activators

Hesperidin and its aglycone metabolite hesperitin (HE) are contained in citrus fruits extracts and peel (Wdowiak et al. 2022), and similarly to the other compounds mentioned it is endowed with the ability to induce SIRT1 (Iside et al. 2020). At the CNS, HE was shown to prevent neuroinflammation in a mouse model of traumatic brain injury and in BV2 cells exposed to oxygen–glucose deprivation followed by recovery, via the SIRT1‐AMPK‐FOXO1 pathway (Song et al. 2022).

Caffeic acid phenethyl ester (CAPE) is contained in honey, olives, apples, artichokes and herbs like oregano. Several studies have proved CAPE's effects by SIRT1‐AMPK induction, mitochondrial safeguard by SIRT1‐PGC1‐α‐DRP1 axis, and modulation of inflammatory pathways via SIRT1‐FOXO1‐Nrf2 and SIRT1‐eNOS‐NF‐kB in models ranging from cadmium‐induced neurotoxicity, hypoxia and spinal cord injury, to nephrotoxicity, peritoneal fibrosis and myocardial ischemia (Li et al. 2018; Hao et al. 2020; Tang et al. 2023; Lu et al. 2024; Zhang et al. 2024).

Chlorogenic acid (CGA) is abundant in MeDi foods and coffee is its largest source (Clifford 1999). CGA was reported to directly restore SIRT1 levels in the cortex of neonatal rats exposed to hypoxia‐ischemia brain injury, targeting Nrf2 and NF‐kB and reducing glial inflammation while exerting neuroprotection (Zheng et al. 2022). Importantly, the combination of CGA administration with physical exercise for 8 weeks in the APP/PS1 AD mouse was shown to significantly enhance the beneficial effects of each alone, preventing hippocampal inflammation, neurodegeneration and cognitive decline. These effects were associated with hippocampal induction of the SIRT1/PGC‐1α signaling pathway (Shi et al. 2023). These findings are an important example of how the consumption of SIRT1 foods can act in synergy with other pillars of a healthy life such as, in this case, physical activity. Other SIRT1‐mediated protective effects of CGA include rescue of mitochondrial functions in oxidized low‐density lipoprotein‐treated endothelial cells in vitro (Tsai et al. 2018), and alleviated isoproterenol‐induced cardiac hypertrophy in H9c2 cells derived from embryonic rat heart ventricles (Ping et al. 2024).

### 6.7 Implications and Key Insights

Overall, it appears evident that many MeDi‐characterizing foods are rich in polyphenols that share the ability to induce SIRT1. Although the neuroprotective effects of several dietary compounds, including resveratrol and quercetin, commonly referred to as “sirtfoods” are attributed to SIRT1 activation, evidence suggests that other members of the sirtuin family may also contribute to these mechanisms. For example, SIRT3, the main mitochondrial deacetylase, plays a crucial role in regulating mitochondrial homeostasis and antioxidant defense via the SIRT1/SIRT3‐FOXO3a axis, processes that are critically altered during the early stages of AD (Wan et al. 2024). Recent experimental studies in a different neurodegenerative disease model, such as ALS, have shown that restoring sirtuins' expression (SIRT3‐SIRT5) contributes to the rescue of mitochondrial respiration (Magri et al. 2024). Moreover, SIRT6 may also participate in flavonoid‐mediated modulation of metabolic and stress‐response pathways (Deniz et al. 2022). In the context of early AD, where mitochondrial dysfunction and metabolic alterations represent early pathogenic events, the multiple sirtuins' modulation can be an important way through which diet‐derived molecules exert neuroprotective effects.

While preclinical models offer a plethora of encouraging data on the resulting beneficial effects on the CNS, translation into actual human benefits faces several challenges. First, the human brain environment is far more complex than any animal model. In addition, the actual human diet cannot be compared to the controlled settings of animal or in vitro experimentation. Another major drawback is the often low bioavailability of each component. In this regard, significant advances have been driven by progress in pharmaceutical technology. New delivery systems have been developed that can improve bioavailability by addressing chemical and biological issues mainly responsible for limited absorption. The aim is to protect molecules from degradation in the GI tract, improve solubility and directly enhance permeability through biological membranes. Nanocarriers include liposomes, nanoemulsions and polymer nanoparticles. More recently, advanced generations of vesicular carriers have been developed, including transferosomes, ethosomes, invasomes and virosomes, optimized for different routes of delivery or targets (Doroshenko and Shevchenko 2026). Intranasal administration of lipid carriers is particularly valuable for direct delivery to the brain through trigeminal and olfactory nerves (Mubarak et al. 2025). Emerging multilamellar nanocarriers such as vesosomes and spongosomes are optimized for controlled and site‐specific release (Doroshenko and Shevchenko 2026). The inclusion in nanoformulations has been explored for some relevant SIRT1‐inducing MeDi compounds, mainly resveratrol but also quercetin, hesperidin and naringenin, proving in general increased bioavailability and enhanced beneficial effects (Andrade et al. 2018; Locatelli et al. 2018; Fakhri et al. 2022; Hoang et al. 2026). In the last few years, specialized vesicular systems called phytophospholipid complexes or “phytosomes” have also been developed. Rather than encapsulating systems, these are direct complexations of phytochemicals, like polyphenols and flavonoids, with phospholipid polar heads. The advantage is represented by increased solubility, stability and membrane permeability, with superior systemic and localized delivery. Data are available for phytosomes carrying different compounds including quercetin, resveratrol and naringenin (Lu et al. 2019; Nesci et al. 2023; Liu, Barber, et al. 2025). Therefore, the scenario for the development of innovative formulations appears very rich and promising and will certainly contribute to widen the use of these natural compounds.

Finally, it is reasonable to think that a balanced choice of SIRT‐foods and the full adherence to the MeDi could yield a potentiation through synergy. In addition, the cooking technique itself can ameliorate the preservation of polyphenols content (Rinaldi de Alvarenga et al. 2019). The establishment of such dietary‐metabolic network would predictably be capable of mitigating hypoxia‐induced and age‐related mitochondrial dysfunction. Nevertheless, many of the cited compounds are already available in the market as supplements, and new administration routes are under investigation.

## 7 Conclusions and Perspectives

In the present review we have offered an up‐to‐date description of the variety and significance of microglial phenotypes that characterize the onset and development of AD, and their strict correlation with energetics and mitochondrial functionality. We moved to highlight how microglia can mediate the effects of hypoxia, a disease modifying factor that plays a prominent role during early asymptomatic stages as well as in more advanced stages. We have discussed the centrality of SIRT1 in regulating the main pathways involved in the energy‐inflammation axis in this context, and the ensuing neuroprotection largely established in preclinical models. Finally, we have reviewed the evidence on the potential benefits of SIRT‐1 inducing dietary polyphenols present in the MeDi, which account for the neuroprotective role associated with a full adherence to this diet. This offers promising avenues for prevention/intervention against hypoxia and metabolic stress that affect the trajectory of AD development. Future research should aim at mechanistic studies at the microglial level to fully understand the effects of each dietary component, while facing the several translational challenges that remain, from the low bioavailability and variable pharmacokinetics to the need for well‐designed clinical studies. Finally, the development of effective and targeted delivery systems is an exciting new direction of research in the field.

## Author Contributions

**Sara Merlo:** conceptualization, funding acquisition, writing – original draft, writing – review and editing. **Cristiana Lucia Rita Lipari:** conceptualization, writing – original draft, writing – review and editing. **Aurora Patti:** conceptualization, writing – original draft. **Maria Angela Sortino:** conceptualization, funding acquisition, writing – original draft, writing – review and editing.

## Funding

This work was supported by the University of Catania Pia.ce.ri Linea 2 (INTERACTIVE) to M.A.S. (#20722142204) and to S.M. (#57722172152) and University of Catania Pia.ce.ri Linea 3 Starting Grant (EMINENT, #57722172146) to S.M.

## Conflicts of Interest

The authors declare no conflicts of interest.

## Data Availability

Data sharing not applicable to this article as no datasets were generated or analysed during the current study.
